# Prevention of SARS-CoV-2 Infection Among Police Officers in Poland—Implications for Public Health Policies

**DOI:** 10.3390/ijerph17239072

**Published:** 2020-12-04

**Authors:** Filip Raciborski, Mateusz Jankowski, Mariusz Gujski, Jarosław Pinkas, Piotr Samel-Kowalik, Artur Zaczyński, Igor Pańkowski, Kamil Rakocy, Waldemar Wierzba

**Affiliations:** 1 Department of Prevention of Environmental Hazards and Allergology, Medical University of Warsaw, 02-091 Warsaw, Poland; filip.raciborski@wum.edu.pl (F.R.); piotr.samel@wum.edu.pl (P.S.-K.); 2 School of Public Health, Centre of Postgraduate Medical Education, 01-826 Warsaw, Poland; mjankowski@cmkp.edu.pl (M.J.); jpinkas@cmkp.edu.pl (J.P.); 3 Central Clinical Hospital of the Ministry of the Interior and Administration in Warsaw, 02-507 Warsaw, Poland; artur.zaczynski@cskmswia.pl (A.Z.); igor.pankowski@cskmswia.pl (I.P.); waldemar.wierzba@cskmswia.pl (W.W.); 4 KR Consulting, 00-001 Warsaw, Poland; rakkam@gmail.com; 5 UHE Satellite Campus in Warsaw, University of Humanities and Economics in Łódź, 01-513 Warsaw, Poland

**Keywords:** coronavirus, COVID-19, SARS-CoV-2, infection prevention, police

## Abstract

Background: This study aimed to characterize sources of knowledge on the means of prevention of SARS-CoV-2 infections as well as to assess the methods of preventing SARS-CoV-2 infection among police employees in Poland and their potential impact on the risk of SARS-CoV-2 infection. Methods: The study consisted of two phases: questionnaire and laboratory tests for SARS-CoV-2 infection. The questionnaire included 30 questions related to risk factors, knowledge about SARS-CoV-2, and methods of infection prevention. Results: Data were obtained from 5082 police employees. The most common source of knowledge for a daily update on SARS-CoV-2 infection prevention was the Internet (42.6%), television (40.3%), and radio (39.7%). The most commonly used methods of SARS-CoV-2 infection included washing one’s hands for at least 20 s (95.8%), wearing facemasks (82.9%), and physical distancing (74.9%). Results of IgG tests were lower in police units where the overall compliance with the preventive measures was higher (*p* < 0.01). Women were more likely to exercise SARS-CoV-2 infection prevention behaviors compared to men. Compliance with the recommended protective measures increased with age. Conclusions: Lower anti-SARS-CoV-2 IgG seropositivity rates were observed in police units with better overall compliance with the preventive measures, suggesting the key importance of group rather than individual behaviors.

## 1. Introduction

Coronavirus Disease 2019 (COVID-19) is an acute infectious disease of the respiratory system caused by severe acute respiratory syndrome coronavirus 2 (SARS-CoV-2) infection [1,2]. The first cases of COVID-19 appeared in early December 2019 in Wuhan, Hubei Province, China [3]. On 11 March 2020, the World Health Organization (WHO) declared the COVID-19 outbreak a global pandemic [4]. As of 7 September 2020, nearly 27 million cases of COVID-19 have been reported worldwide, including 876,616 deaths [5]. COVID-19 cases in Europe account for 17% of those globally.

The clinical presentation of SARS-CoV-2 infection is highly variable, ranging from asymptomatic to a severe or critical course [6,7,8]. The predominant group of COVID-19 patients (81%) manifests with only mild to moderate symptoms [6,7]. Usually, symptoms appear within 2 to 14 days after exposure to the virus [9]. However, a growing number of publications show that approximately 45% of COVID-19 cases may be asymptomatic [10]. The most common symptoms of SARS-CoV-2 infection include fever, dry cough, and shortness of breath [8]. Clinical manifestation of SARS-CoV-2 infection also includes non-respiratory symptoms, such as gastrointestinal (diarrhea, nausea, lack of appetite) and neurological symptoms (olfactory or taste disorders) [11,12,13]. It is estimated that 10% to 20% of COVID-19 cases require hospitalization [14]. The case fatality rate has been estimated to be between 0.5% and 2.4% [15,16]. The risk factors for COVID-19-related death include older age; underlying medical conditions, such as hypertension, diabetes mellitus, cancer, and obesity; and may also vary depending on gender and ethnicity [17,18]. 

According to the WHO guidelines, a COVID-19 diagnosis is based on the detection of SARS-CoV-2 RNA by real-time reverse transcription–polymerase chain reaction (RT-PCR) [19]. Moreover, serological tests for SARS-CoV-2 antibodies are available for epidemiological purposes [20]. Serological assays are based on the detection of various classes of immunoglobulins (Ig) against SARS-CoV-2 (mostly on IgA, IgM, or IgG) using enzyme-linked immunosorbent assays (ELISA), chemiluminescent microparticle immunoassays (CLIA), or immunometric assays [20,21].

The primary SARS-CoV-2 transmission mode is person-to-person contact through respiratory droplets generated by coughing, sneezing, or speaking [22,23]. Moreover, transmission through direct contact with an infected subject or indirect contact via contaminated surfaces was also observed [23]. Additionally, some studies suggest that SARS-CoV-2 may be transmitted via airborne transmission or transmission through aerosols [23]. SARS-CoV-2 transmission can be particularly high in crowded and confined indoor spaces [24].

To mitigate the spread of SARS-CoV-2 infection, the WHO put forward a strategic preparedness and response plan that included public health measures that should be incorporated into the national public health policies aimed at the response to the COVID-19 pandemic [25]. A meta-analysis including 172 studies from 16 countries showed that physical distancing of at least 1 m, face masks, eye protection, and hand hygiene are the most effective ways of preventing SARS-CoV-2 transmission in community settings [26,27]. Due to the lack of a vaccine as well as specific treatment methods, the abovementioned preventive measures are crucial to limit SARS-CoV-2 transmission. 

Available data regarding SARS-CoV-2 transmission showed that work related transmission contributes significantly to the epidemic outbreak [28,29,30]. The occupational groups with the highest risk of SARS-CoV-2 transmission include healthcare workers, transport workers, services and retail workers, cleaning service workers, and public safety workers, such as police officers or firefighters [29]. 

Most studies on occupational exposure are focused on healthcare workers [30,31,32]. In Poland, the Police task force are an occupational group that was also actively involved in combating the COVID-19 pandemic. The Polish Police was tasked with daily monitoring of people in quarantine or self-isolation. People in mandatory quarantine were obligated to stay home and police officers were tasked with daily monitoring of compliance to the quarantine [33]. It is estimated that Polish Police have carried out more than 10 million visits among people in quarantine or self-isolation [34]. Moreover, Polish Police were actively involved in the protection of public gatherings. Due to the high number of interpersonal contacts associated with occupational exposure, police workers were at high risk of infection. Preventive strategies for high-risk occupational groups are crucial to limit SARS-CoV-2 transmission in the community.

This study aimed to characterize sources of knowledge on the means of prevention of SARS-CoV-2 infections as well as to assess the methods of preventing SARS-CoV-2 infection among police employees in Poland (officers and civil workers) and their potential impact on the risk of SARS-CoV-2 infection.

## 2. Materials and Methods

### 2.1. Study Design and Population

This cross-sectional survey was carried out between 22 June and 8 July 2020 among police employees (officers and civilians) from the Mazowieckie Province, Poland. The study consisted of two phases—a questionnaire and laboratory tests for current (RT-PCR) and previous (ELISA) SARS-CoV-2 infection.

The questionnaire prepared for this study included 30 questions related to risk factors, knowledge about SARS-CoV-2, and methods of infection prevention. Moreover, questions related to the socioeconomic characteristics and the types of occupational activities were also addressed. The questionnaire was based on the previously published COVID-19-oriented research, with special emphasis on the WHO guidebook on behavioral insights studies related to COVID-19 [35]. A pilot test of the first draft of the questionnaire was carried out among 10 police employees. After the pilot test, two questions related to the type of service were added and 1 question related to the personal characteristics was removed due to the potential possibility of identifying individual respondents. The questionnaires were collected using a Computer-Assisted Web Interview (CAWI) method. The survey takes no more than 15 minutes. Field control was enabled to avoid missing data.

For laboratory testing, nasopharyngeal swabs and serum samples were collected. Detection of SARS-CoV-2 RNA by RT-PCR qualified as a positive test result (current infection). Anti-SARS-CoV-2 IgM_+_IgA index above 8 was considered positive, while participants with indexes between 6 and 8 were considered equivocal. Anti-SARS-CoV-2 IgG index above 6 was considered positive and participants with indexes between 4 and 6 were considered equivocal. All the testing procedures were carried out in accordance with the WHO guidelines. A detailed testing methodology was described in the previous article [36].

The RT-PCR tests for the detection of SARS-CoV-2 RNA (the Orf1ab and N target gene regions) were carried out with the DiaPlexQ™ Novel Coronavirus (2019-nCoV) Detection Kit (SolGent Co., Ltd.; Daejeon, South Korea) (accuracy 95–99%) [37].

The antibodies tests targeting SARS-CoV-2-specific antigens, spike glycoprotein (S), and nucleocapsid protein (N) were carried out with COVID-19 ELISA IgG and IgM_+_IgA kits (Vircell S.L., Granada, Spain) (sensitivity: IgM_+_IgA 88%; IgG 85%; specificity: IgM_+_IgA 99%; IgG 98%) [38]. All samples were collected by a trained medical staff using transport sets specially designed for collecting clinical material for the diagnosis of SARS-CoV-2 infection.

This study was carried out on an effective random sample of 5082 police employees from the Mazowieckie Province, Poland. A random sample selection was ensured by using the group selection technique (with the probability of drawing proportional to the size of the group) and stratified selection. Participation in the study was voluntary. The study protocol was approved by the Ethical Review Board at the Medical University of Warsaw, Warsaw, Poland (document no. KB/87/2020).

### 2.2. Variables

The scale for the assessment of compliance with the measures aimed at preventing SARS-CoV-2 infection was based on 8 questions regarding: (i) hand washing; (ii) avoidance of touching one’s nose and mouth; (iii) the use of hand disinfectants; (iv) caution when opening mail; (v) wearing face masks; (vi) physical distancing (at least 2 m); (vii) surface disinfection; and (viii) phone disinfection. Other behaviors not directly related with the reduction of SARS-CoV-2 infection risk (e.g., covering one’s mouth, flu vaccinations) were not taken into account. A score of 2 was awarded if a subject practiced a particular form of prevention at the time of the study; a score of 1 was awarded when the subject used to practice that form before (and discontinued it). Zero points were awarded for non-compliance with a particular preventive measure. The scale was an additive scale with the total score ranging from 0–16 points. The scale reliability test afforded a Cronbach’s alpha of 0.720.

The scale for the assessment of the use of information sources was based on 8 questions regarding the frequency of using the following sources when searching for information on the novel coronavirus: (i) TV; (ii) radio; (iii) press; (iv) conversations with family and relatives; (v) conversations with friends; (vi) websites/news; (vii) social media; and (viii) official government announcements. For each source, the subjects chose one of 6 possible answers ranging from “never” to “several times a day”. A scale of 0 to 40 points was constructed by adding individual scores (from 0 to 5) assigned to each answer. The scale reliability test afforded a Cronbach’s alpha of 0.848.

Each subject was asked a series of questions regarding the prevalence of chronic diseases. Diseases taken into account included respiratory diseases, allergies, urinary tract diseases, cardiovascular diseases, diabetes, gastrointestinal tract diseases, endocrine diseases, and cancer.

External data regarding the number of registered cases and deaths per 10,000 inhabitants of individual districts in the Mazovian voivodeship were also included in the logistic regression models. Epidemiological data were derived from the reports published by the State Sanitary Inspection (as of 8 July 2020).

### 2.3. Statistical Analysis 

Statistical analysis was carried out using the SPSS software package (IBM, Armonk, NY, USA) version 26. Chi-square test was used for the determination of significance for ordinal and categorical variables. Statistical significance was defined as *p* < 0.05. The scales were verified using scale reliability analysis. The reliability of the scales was assessed using Cronbach’s alpha. The powers of relationship between the variables were assessed using odds ratios (OR) as calculated from multivariate logistic regression models.

Model I. The explained variable consisted of a positive or an ambiguous (i.e., ≥4) result of the anti-SARS-CoV-2 IgG screening test. Explanatory variables were entered as a series of dummy variables (0–1) and included: gender, age, population of the area of residence, living alone or with others, type of service (officer vs. civilian employee), type of work, daily number of contacts with other people, leaving the country to visit selected destinations since 1 January 2020, as well as the eight preventive measures to minimize the risk of novel coronavirus infection. Continuous variables entered into the model included the number of infection cases per 10,000 district inhabitants, the number of infection-related deaths per 10,000 district inhabitants, and the percentage of subjects within the particular police unit presenting with a positive or ambiguous result of anti-SARS-CoV-2 antibody tests.

Model II. The explained variable produced a score of 13–16 using the scale of compliance with the preventive measures aimed at reducing the risk of SARS-CoV-2 infection (0–16 points). Explanatory variables were entered as series of dummy variables (0–1) and included: gender, age, population of the area of residence, living alone or with others, type of service (officer vs. civilian employee), type of work, daily number of contacts with other people, prevalence of at least chronic variables included in the list, self-assessed health status, and frequency of using various sources of information regarding the novel coronavirus. Continuous variables entered into the model included the number of infection cases per 10,000 district inhabitants, the number of infection-related deaths per 10,000 district inhabitants, and the overall score in the scale assessing the use of coronavirus information sources.

## 3. Results

### 3.1. Group Characteristics

Police officers accounted for 79.2% of the study population of 5082 subjects, whereas civilian employees accounted for the remaining 20.8%. Female subjects accounted for 33.5% of the study population. The mean age of subjects was 39.6 years (SD = 8.9) for the overall population and 40.7 years (9.6) and 39 years (8.5) for female and male subjects, respectively.

A total of 30.1% of subjects were engaged only in office work, while another 17.3% were engaged in field service alone. The remaining subjects (52.6%) were engaged in both types of work. Among all participants, 2.4% received flu shots during the previous influenza season.

### 3.2. Sources of Knowledge about SARS-CoV-2 Infection Prevention

A vast majority of the overall population of police workers (95.6%) agreed with the statement that they were well informed on the SARS-CoV-2 coronavirus. In the group of police officers, 23.0% of subjects agreed with the statement whereas 72.4% rather agreed with it. In the group of civilian employees, the respective percentages were 18.0% and 78.3% (*p* < 0.01).

The most common source of knowledge for a daily update on SARS-CoV-2 infection prevention was the Internet (websites), but also traditional media, such as television and radio. Almost one third of participants (31.1%) followed daily announcements published by the official government institutions. Among participants, 28.5% used social media to search for information about SARS-CoV-2 infection daily. Every fourth respondent talked with family or friends about the coronavirus daily. Newspapers were the least common source of knowledge about the coronavirus. Details are presented in Table 1.

### 3.3. Prevalence of SARS-CoV-2 Antibodies

No active SARS-CoV-2 infection was detected by means of RT-PCR in any of the tested subjects. A positive result of the IgG screening test (>6) was determined in 4.3% of subjects. In another 13.2% of subjects, ambiguous results (range of 4–6) were obtained. A positive result of the IgA + IgM screening test (>8) was determined in 8.9% of subjects. In another 9.8% of subjects, ambiguous results (range of 6–8) were obtained. Detailed data on the infection rates within the group of police workers are provided in the previously published paper [36].

### 3.4. Descriptive Analyses

#### 3.4.1. SARS-CoV-2 Infection Prevention Methods

**Figure 1:**
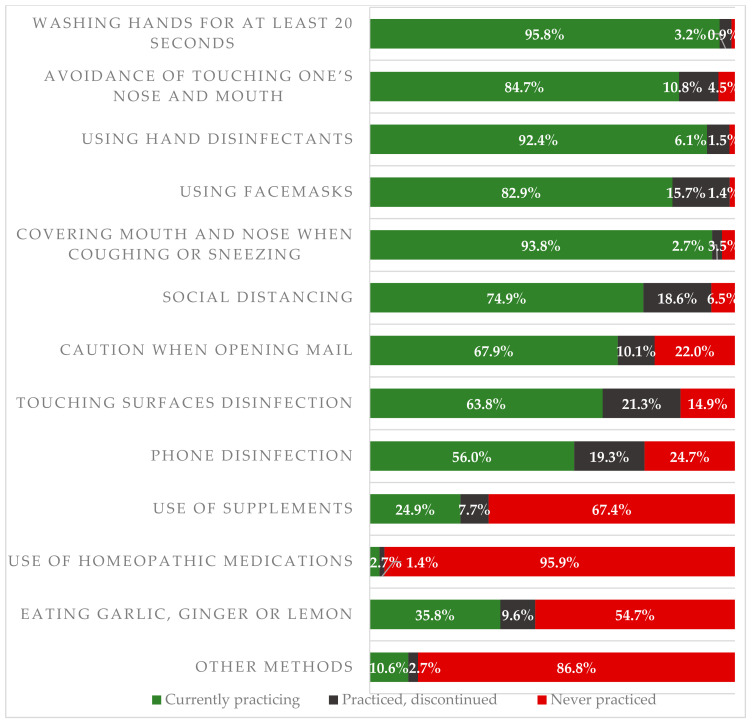
Methods of SARS-CoV-2 infection prevention practiced by the study subjects.

In the study group (*n* = 5082), the most commonly used methods of SARS-CoV-2 infection included washing one’s hands for at least 20 seconds and covering one’s nose and mouth while coughing or sneezing. These behaviors were reported by 95.8% and 93.8% of responders, respectively. Wearing face masks was reported by 82.9% of subjects, while physical distancing was declared by 74.9%. Previous practicing (and subsequent discontinuation) of physical distancing and wearing face masks was declared by 18.6% and 15.7% of subjects, respectively. Detailed data are presented in Figure 1.

**Figure 2:**
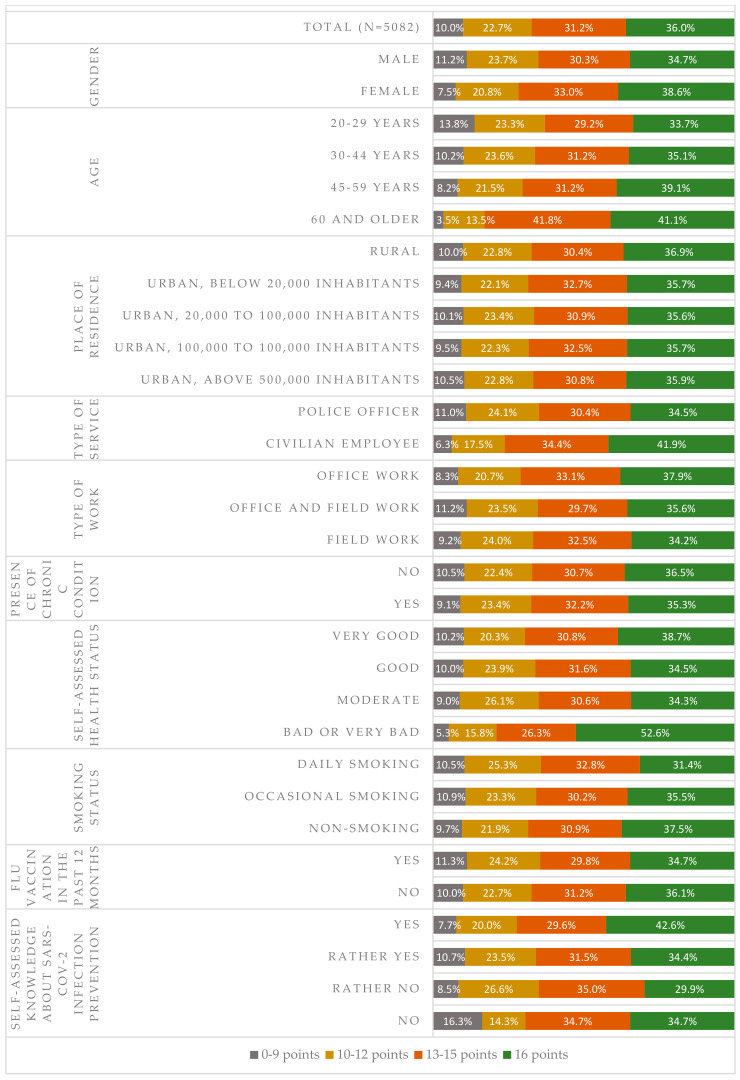
Compliance with the preventive measures aimed at reducing the SARS-CoV-2 infection risk (scale of 0 to 16—the higher the score, the better compliance with recommendations) depending on selected sociodemographic parameters.

The mean score on the scale of compliance with the preventive measures aimed at reducing the risk of SARS-CoV-2 infection (0–16) was 13.4 (SD = 2.8). The median score was 14.0. In the overall population, 36.0% of subjects scored the complete number of 16 points, meaning that they practiced all eight preventive measures selected for further analyses at the time of the study. Another 31.2% of subjects scored 13–15 points, meaning that they practiced nearly all the preventive measures. Every 10th subject (10.0%) practiced the preventive measures in a selective manner or did not practice them at all (score of 0–9). Compliance with the prevention principles was declared more frequently by female subjects as compared to male subjects. Scores of 13 or higher were obtained by 71.6% of female responders and 65.1% of male responders (*p* < 0.001). Age was another factor that had a statistically significant impact on answers provided (*p* < 0.001). Scores of 13 or higher were obtained by 62.9% of the youngest police workers (age range 20–29 years) as compared to 82.9% in the oldest age group (60 or older). Lower compliance with the recommendations was observed in police officers as compared to civilian employees. Scores of 13 or higher were obtained, respectively, by 64.9% and 76.2% of subjects in these groups (*p* < 0.001). Significant relationships were also observed with regard to the type of work (office vs. field) (*p* < 0.01), self-assessed health status (*p* < 0.05), traditional smoking status (*p* < 0.05), and level of awareness regarding the novel coronavirus (*p* < 0.001). No differences were observed with regard to the number of inhabitants in the area of residence (*p* = 0.997), prevalence of at least one chronic disease (*p* = 0.283), or flu shots received during the last influenza season (*p* = 0.925). Detailed data are presented in Figure 2.

#### 3.4.2. Analysis of Relationships Between Variables

Cox & Snell R-Squared of 0.044 and Nagelkerke R-Squared of 0.073 were obtained for the logistic regression model aimed at predicting a positive or ambiguous (i.e., ≥4) result of an IgG screening test. Among the analyzed variables, statistically significant differences were identified for: age of 60 years or older (OR = 2.125; 95%CI 1.300–3.475) vs. age of 20-29 years; below 20,000 inhabitants in the area of residence (OR = 1.361; 95%CI 1.063–1.742) and more than 500,000 inhabitants in the area of residence (OR = 1.387; 95%CI 1.122–1.714) vs. rural residence, and particular caution when opening mail being practiced at the time of the study (OR = 0.813; 95%CI 0.666–0.992) vs. no caution when opening mail being practiced at any time. Statistical significance was also observed for the continuous variable corresponding to the percentage of workers at the particular police unit from whom positive or ambiguous results of IgG screening tests were obtained (*p* < 0.001). Details are presented in Table S1.

The percentage of workers of whom positive or ambiguous results of IgG screening tests were obtained was determined for each police unit taking part in the survey. Then, the results were compared with the mean score in the scale assessing compliance with the protective measures aimed at reducing the risk of SARS-CoV-2 infection. The obtained result was suggestive of the infection rate being lower in groups of more compliant subjects, with Pearson’s coefficient amounting to −0.253 (*p* < 0.01).

The other logistic regression model explained the high scores (13–16 points) in the scale assessing compliance with the protective measures aimed at reducing the novel infection risk (scale of 0–16) against selected factors. Cox & Snell R-Squared of 0.109 and Nagelkerke R-Squared of 0.152 were obtained for this model. Women were more likely to follow more recommendations for preventing new infections than men (OR = 1.182; 95%CI: 1.009–1.385). The willingness to comply with various prevention measures increased with subjects’ age. This was most evident in the eldest age group (60 or more years) where the odds ratio against the group of workers aged 20–29 was determined at OR=2.504 (95%CI: 1.467–4.272). Officers were less willing to comply with recommendations compared to civilian employees (OR = 0.648; 95%CI: 0.518–0.810). Subjects who declared daily numbers of contacted individuals as 20–49 (OR = 0.811; 95%CI: 0.659–0.998) or 50–100 (OR = 0.760; 95%CI: 0.586–0.987) were less strict in their attitudes towards preventive measures than individuals with either very high (over 100) or lower numbers (less than 20) of contacts. Frequent use of radio, press, or government announcements as sources of information on the novel coronavirus favored stricter compliance with prophylactic recommendations (OR between 1.326 and 1.915). On the other hand, a negative relationship with compliance was observed for the overall scale assessing the use of coronavirus information sources (0–40 points). No statistical significance was determined for the remaining parameters. Details are presented in Table 2. 

## 4. Discussion

To the authors’ best knowledge, this is one of the first studies on SARS-CoV-2 infection prevention methods and their impact of seroprevalence among uniformed services (police employees). We observed a relatively high level of knowledge about SARS-CoV-2 infection prevention methods among police employees. Most of the participants practiced preventive behaviors, such as handwashing and using facemasks, however significant deficiency was observed with regard to the practice of social distancing. Our findings showed that women were much more likely to exercise SARS-CoV-2 infection prevention behaviors compared to men. Compliance with the recommended protective measures increased with age. 

Our study showed that police employees used both traditional media (radio and television) as well as new media (Internet) to search for daily information about SARS-CoV-2 infection prevention methods. Moreover, almost one third followed the official government announcements. A study from China showed that media coverage can be considered an effective way of mitigating the spread of the COVID-19 pandemic [39]. In Poland, information regarding the epidemiological situation in the country and news about the SARS-CoV-2 coronavirus were available on the headlines of all mass media; therefore, there was wide access to the information. Instructional materials on SARS-CoV-2 infection prevention methods were broadly available across the traditional as well as online media. Moreover, the Ministry of Health published a daily report with the number of new laboratory-confirmed COVID-19 and COVID-19-related deaths as well as a list of subregions with the highest COVID-19 burden [40]. Police employees also received dedicated informative materials related to SARS-CoV-2 infection prevention methods that should be applied on duty [41]. This document included hazard identification, basic principles of protection against COVID-19, personal protective equipment (PPE) characteristics, and methods of deactivation of SARS-CoV-2 [41]. We can hypothesize that due to the multiple sources of information on the prevention of SARS-CoV-2 infections, police officers declared a high level of knowledge about SARS-CoV-2 infection prevention methods. The study conducted among 471 health care workers in Greece showed that a high level of knowledge concerning the SARS-CoV-2 pandemic among health care workers was significantly associated with positive attitudes and practices towards preventive health measures [42]. Similarly, as in our study, the major source of knowledge about COVID-19 among Greek health care workers was TV/radio (69.8%) and the Internet/web pages/blogs (63%) [42]. This observation confirms the need for health communication regarding the coronavirus using traditional media as well as Internet-based communication methods. 

Various public health measures aimed at limiting the spread of SARS-CoV-2 infection were applied across the world [33,43]. However, findings from the meta-analysis showed that physical distancing, wearing a facemask, eye protection, and hand hygiene are the most effective ways of SARS-CoV-2 infection prevention [26]. In our study, the most widely promoted methods of preventing SARS-CoV-2 infections, i.e., hand hygiene, maintaining physical distance, and wearing a facemask, were declared by the vast majority of respondents. However, a quarter of the respondents did not pay attention to keeping their physical distance at the time of the survey and almost a fifth did not wear a face mask. It is estimated that implementation of physical distancing is associated with a 29% reduction in COVID-19 incidence and a 35% reduction in COVID-19 mortality [44]. We can hypothesize that the lack of compliance with physical distancing rules may result from the occupational duties, especially among police officers designated to protecting public gatherings. Compliance with wearing a facemask may be associated with the ergonomic characteristics of different types of facemasks [45,46]. The study on facial skin temperature and discomfort when wearing a facemask showed that wearing N95 respirators compared to surgical masks produces increased facial skin temperature, greater discomfort, and lower wearing adherence [45]. Professional groups required to wear a facemask in the workplace may use a personalized fitting method (e.g., device with a 3-dimensional solution) for prevention of oronasal mask-related pressure ulcers [46].

Moreover, our findings revealed that a significant percentage of police employees did not comply with the principles of mobile phone disinfection and touching surfaces. A systematic review including 56 papers showed that mobile phones represent a significant pathway for microbial transmission in the healthcare setting as well as in community settings [47]. It is suggested that surfaces of mobile phones may play an important role in the transmission of SARS-CoV-2 infections in an epidemic outbreak [47]. Disinfecting objects and surfaces is a part of a strategy aimed at mitigating the spread of SARS-CoV-2 infection [48,49]. Given that the study looked at police employees who should be considered a high-risk group, the observed shortcomings in compliance with SARS-CoV-2 infection prevention guidelines require urgent educational actions. The study conducted among 123,768 large labor-intensive factory workers in China showed that the majority of respondents had a strong awareness of COVID-19, however some knowledge misconceptions (e.g., related to garlic and Vitamin C in infection prevention) were also observed [50]. Moreover, better-educated respondents had increased levels of knowledge and practices related to COVID-19 [50]. In our study, eating garlic, ginger, or lemon was practiced by 35.8% of respondents. This observation suggests a need to provide an evidence-based educational campaign to combat the spread of misinformation on SARS-CoV-2 prevention methods.

Women were more likely to practice preventive behaviors than men. This is in line with previous observations related to preventive behaviors during the COVID-19 pandemic. The study conducted in China after the lockdown of Hubei Province showed that women displayed a higher level of knowledge about SARS-CoV-2 infection prevention than men [51]. Moreover, women more often practiced behaviors such as wearing a facemask and maintaining social isolation than men [51]. Additionally, numerous studies showed that gender is a determinant of compliance with handwashing recommendations and impacts the frequency of handwashing [52,53]. Our findings are in line with the study carried out in a sample of 2323 Polish secondary school students [54], where female secondary school students presented a higher level of knowledge about SARS-CoV-2 infection prevention. Similarly, as in our study, females practiced hand hygiene and personal protection behaviors more often than men [54].

Our findings showed that police employees aged 60 years and more presented higher compliance with SARS-CoV-2 infection prevention methods compared to the younger groups. Older adults are at higher risk for developing more serious complications from COVID-19 [55]. We can hypothesize that older police employees were afraid of the potential health consequences of SARS-CoV-2 infection and more often implemented preventive methods than younger groups because of that. Non-compliance with the recommended protective measures among adolescents and young adults was noted by the WHO, who asked the adolescents and young adults to follow the recommendations aimed at preventing SARS-CoV-2 transmission in the community [56]. An asymptomatic or oligosymptomatic COVID-19 course is observed among younger age groups [6,7] and due to this, they may underestimate the threat of COVID-19 to their own health, while becoming a source of transmission of the virus to their parents and grandparents, who may be severely affected.

In our study, people using more reliable/institutionalized sources of knowledge about the coronavirus (i.e., radio, press, official announcements) showed a higher level of compliance with infection prevention measures. An excessive amount of information, including that of questionable reliability, is conducive to departing from the principles of infection prevention. The COVID-19 pandemic is the first pandemic occurring in the era of social media. Social media allows for the quick provision of information on SARS-CoV-2 infection prevention, but may also be a source of misinformation. Misinformation on COVID-19 may shape individual’s response, increasing the risk of hazardous behaviors [57,58]. Healthcare professionals are responsible for providing evidence-based knowledge about SARS-CoV-2 infection prevention. In Poland, the family doctor has the highest level of trust of all medical professions [59], so family doctors should be tasked with disseminating information about evidence-based preventive methods to limit the transmission of SARS-CoV-2 infection.

Among the infection prevention methods analyzed in the logistic regression model, a statistically significant relationship between the risk of a positive or ambiguous result of IgG screening test was observed only for caution when opening mail. The authors are inclined towards a hypothesis that this finding is not necessarily suggestive of the efficacy of this particular preventive measure; instead, it may mean that individuals declaring this behavior during the study tended to more strictly comply with other recommendations as well. Thus, this measure can be considered an indicator of one’s attitude to the prevention of new SARS-CoV-2 infections. 

With the exception of one situation, no relationships were observed between compliance with prevention methods and positive or ambiguous history of infection (IgG). Results of IgG screening tests were lower in police units where the overall compliance with the preventive measures was higher. Lack of relationships between compliance with most measures aimed at preventing SARS-CoV-2 infection and the positive or ambiguous IgG screening test results (which confirmed the history of infection) at the level of individual subjects may suggest that the problem of infection prevention should be considered from the perspective of groups rather than individuals. In other words, in the case of non-complying individuals, the risk may be markedly reduced by proper behavior of other individuals in their close surroundings. The phenomenon is similar to that observed in relation to vaccinations and herd immunity. The aptness of this hypothesis is supported by the result of ecological assessment in which a relationship was demonstrated between the degree of compliance with the preventive measures at the level of police units and the incidence of positive or ambiguous IgG screening test results among the employees of these units. This may be indicative of the key importance of group rather than individual behaviors when it comes to preventing new infections. Public health policies aimed at mitigating spread of SARS-CoV-2 infection should include group behaviors.

Our findings have some practical implications. Lower compliance with SARS-CoV-2 infection prevention methods among men and younger age groups points to the need for education on the methods of preventing SARS-CoV-2 infections in these groups. In clinical practice, physicians, especially family doctors, should make sure to educate on SARS-CoV-2 prevention methods, especially among the high-risk groups, such as the uniformed services. Employers should promote group preventive behaviors as such measures have the greatest effects on limiting SARS-CoV-2 transmission in occupational settings. Methods of SARS-CoV-2 infection prevention should be included in employee training and work regulations. 

Most studies concerning occupational exposure to the SARS-CoV-2 coronavirus were carried out among healthcare workers [28,29,30,31,32]. Data on preventive behaviors among other occupational groups is very limited. Due to the limited scientific evidence, comparing our results to other studies is very difficult. The results of our study emphasize the urgent need for further research on the topic in other occupational groups at the highest risk of SARS-CoV-2 transmission, including transport workers, services and retail workers, as well as uniformed services [29]. 

This study has several limitations. Firstly, the practiced preventive behaviors were self-declared by the participants, but were not verified by the research team, so we cannot exclude the possibility of recall bias. Secondly, we did not assess the frequency of practiced preventive behaviors. We cannot rule out that some of the participants exercising the SARS-CoV-2 preventive methods may have done it too rarely. Nevertheless, this is the first study on SARS-CoV-2 infection prevention among police employees in Poland. Thirdly, this study was carried out among uniformed services and results cannot be generalized to the whole population.

## 5. Conclusions

Lower anti-SARS-CoV-2 IgG seropositivity rates were observed in police units with better overall compliance with the preventive measures, suggesting the key importance of group rather than individual behaviors when it comes to preventing new infections; such group behaviors should be included in public health policies aimed at mitigating SARS-CoV-2 transmission. Willingness to comply with a larger number of preventive methods increased with age. The group of subjects aged 60 or older was most willing to comply with the recommendations for the prevention of SARS-CoV-2 infection. Individuals seeking information on the novel coronavirus in the more institutionalized sources, such as radio, press, or TV, showed better compliance with the recommended prevention methods.

## Figures and Tables

**Table 1:** Source of knowledge on SARS-CoV-2 infection prevention (*n* = 5082).

	The Frequency of Using Individual Sources of Knowledge on SARS-CoV-2 Infection Prevention *n* = 5082					
Source of Knowledge	Several Times a Day	Daily	Several Times a Week	Once a Week	Less than Once a Week	Never
Television	886 (17.4%)	1164 (22.9%)	1467 (28.9%)	621 (12.2%)	643 (12.7%)	301 (5.9%)
Radio	1167 (22.7%)	866 (17.0%)	1411 (27.8%)	434 (8.5%)	707 (13.9%)	497 (9.8%)
Newspapers	183 (3.6%)	350 (6.9%)	564 (11.1%)	588 (11.6%)	1115 (21.9%)	2282 (44.9%)
Family	748 (14.7%)	610 (12.0%)	1770 (34.8%)	784 (15.4%)	889 (17.5%)	281 (5.5%)
Friends	688 (13.5%)	586 (11.5%)	1791 (35.2%)	821 (16.2%)	929 (18.3%)	267 (5.3%)
Websites/news	1095 (21.5%)	1073 (21.1%)	1469 (28.9%)	572 (11.3%)	565 (11.1%)	308 (6.1%)
Social media	759 (14.9%)	687 (13.5%)	1096 (21.6%)	501 (9.9%)	764 (15.0%)	1275 (25.1%)
Official government announcements	528 (10.4%)	1053 (20.7%)	1430 (28.1%)	808 (15.9%)	918 (18.1%)	345 (6.8%)

**Table 2:** Relationship between compliance with most preventive measures aimed at reducing the SARS-CoV-2 infection risk and selected parameters. Logistic regression model.

Variable	OR	95%CI OR	
		Lower	Upper
Gender: female	**1.182**	**1.009**	**1.385**
Age: 30–44 years	**1.366**	**1.124**	**1.661**
Age: 45–59 years	**1.660**	**1.308**	**2.108**
Age: 60 and older	**2.504**	**1.467**	**4.272**
Urban, below 20,000 inhabitants	1.015	0.826	1.248
Urban, 20,000 to 500,000 inhabitants	0.953	0.804	1.129
Urban, above 500,000 inhabitants	0.958	0.806	1.139
Living together with 1 or more other individuals	0.936	0.736	1.190
Police officer	**0.648**	**0.518**	**0.810**
Office work	0.934	0.749	1.165
Office and field work	0.898	0.749	1.076
Daily number of contacts: 10–19 individuals	0.896	0.725	1.108
Daily number of contacts: 20–49 individuals	**0.811**	**0.659**	**0.998**
Daily number of contacts: 50–100 individuals	**0.760**	**0.586**	**0.987**
Daily number of contacts: >100 individuals	0.682	0.430	1.082
Prevalence of chronic diseases	0.954	0.829	1.099
Self-assessed health status: very good	0.926	0.289	2.971
Self-assessed health status: good	0.702	0.220	2.242
Self-assessed health status: moderate	0.540	0.166	1.752
TV: once or several times a week	0.951	0.776	1.166
TV: once or several times a day	1.085	0.860	1.369
Radio: once or several times a week	**1.434**	**1.183**	**1.737**
Radio: once or several times a day	**1.326**	**1.076**	**1.634**
Press: once or several times a week	**1.339**	**1.132**	**1.585**
Press: once or several times a day	**1.428**	**1.105**	**1.845**
Conversations with relatives: once or several times a week	1.037	0.819	1.313
Conversations with relatives: once or several times a day	1.164	0.828	1.636
Conversations with friends: once or several times a week	0.958	0.757	1.213
Conversations with friends: once or several times a day	1.060	0.750	1.498
Websites: once or several times a week	1.025	0.826	1.271
Websites: once or several times a day	1.032	0.817	1.305
Social media: once or several times a week	1.084	0.911	1.290
Social media: once or several times a week	0.932	0.763	1.138
Government announcements: once or several times a week	**1.356**	**1.143**	**1.607**
Government announcements: once or several times a day	**1.915**	**1.555**	**2.359**
Information source use assessment scale (0–40)	**0.828**	**0.809**	**0.848**
Cases per 10,000 inhabitants.	0.988	0.962	1.014
Deaths per 10,000 inhabitants.	1.104	0.842	1.446
Constant *	8.857		

* Reference category: male; age: 20–29 years; place of residence: rural; civilian employee; field work; daily number of contacts: <10 individuals; no chronic diseases; self-assessed health status: bad or very bad; TV: never or less than once a week; radio: never or less than once a week; press: never or less than once a week; conversations with relatives: never or less than once a week; conversations with friends: never or less than once a week; websites: never or less than once a week; social media: never or less than once a week; government announcements: never or less than once a week. Bold format reffers to statisticaly significant results.

## Supplementary Materials

The following are available online at https://www.mdpi.com/1660-4601/17/23/9072/s1, Table S1: Correlation between a positive or ambiguous result of the IgG screening test and the selected risk factors. Logistic regression model.
