# Supplementary material for: Prevention of SARS-CoV-2 Infection Among Police Officers in Poland—Implications for Public Health Policies
Source: Int J Environ Res Public Health. 2020 Dec 4;17(23):9072. doi: 10.3390/ijerph17239072 (PMC7731213; doi:10.3390/ijerph17239072)
Supplement: Supplementary file 1 [file ijerph-17-09072-s001.pdf]

Supplementary Material

Table S1. Correlation between a positive or ambiguous result of the IgG screening test and the selected risk factors. Logistic regression model.

	OR	95%CI OR	
		**Lower**	**Upper**
ABM_2_2__Female	1.168	0.969	1.407
rABM_1_30_44	1.245	0.977	1.586
rABM_1_45_59	1.152	0.872	1.522
rABM_1_60	2.125	1.300	3.475
rABM_3_urban_below_20	1.361	1.063	1.742
rABM_3_urban_20_500	1.227	0.997	1.510
rABM_3_urban_above_500	1.387	1.122	1.714
ABM_4_2__Living_with_1_or_more_individuals	1.261	0.936	1.699
ABM_6_1__Police_officer	1.080	0.834	1.398
ABM_8_1__Office only__incl_management_	1.001	0.769	1.304
ABM_8_2__Both_office_and_field	0.896	0.720	1.116
ABM_9_1__Below_5_individuals	0.918	0.401	2.101
ABM_9_2__5_9_individuals	0.877	0.494	1.558
ABM_9_3__10_19_individuals	1.079	0.630	1.848
ABM_9_4__20_49_individuals	1.081	0.633	1.846
ABM_9_5__50_100_individuals	0.982	0.556	1.735
ABM_25_1__Yes	0.769	0.449	1.317
Cases_10k	1.009	0.978	1.041
Number_deaths_10k	0.939	0.683	1.289
ABM_28_1_1__Practicing_now	0.731	0.335	1.598
ABM_28_2_1__Practicing_now	1.145	0.766	1.713
ABM_28_3_1__Practicing_now	1.562	0.759	3.213
ABM_28_9_1__Practicing_now	0.813	0.666	0.992
ABM_28_11_1__Practicing_now	0.987	0.510	1.908
ABM_28_12_1__Practicing_now	0.990	0.719	1.362
ABM_28_14_1__Practicing_now	1.229	0.933	1.619
ABM_28_15_1__Practicing_now	1.057	0.841	1.330
ABM_28_1_2__Practiced_but_not_any_more	0.693	0.289	1.662
ABM_28_2_2__Practiced_but_discontinued	1.242	0.796	1.938
ABM_28_3_2__Practiced_but_discontinued	2.002	0.925	4.330
ABM_28_9_2__Practiced_but_discontinued	0.848	0.635	1.132
ABM_28_11_2__Practiced_but_discontinued	0.951	0.482	1.876
ABM_28_12_2__Practiced_but_discontinued	0.965	0.678	1.372
ABM_28_14_2__Practiced_but_discontinued	1.175	0.879	1.570
ABM_28_15_2__Practiced_but_discontinued	1.000	0.774	1.292
rLAB_2_3_mean	736.854	281.071	1931.735
Constant	0.022		
